# Serum Calprotectin Concentration in Patients with Oral Lichen Planus: A Cross-Sectional Study

**DOI:** 10.3390/medicina62050891

**Published:** 2026-05-06

**Authors:** Ana Glavina, Helena Erić, Daniela Šupe-Domić, Antonija Tadin

**Affiliations:** 1 Department of Dental Medicine, University Hospital of Split, 21000 Split, Croatia; atadin@mefst.hr; 2 Department of Oral Medicine, Study of Dental Medicine, School of Medicine, University of Split, 21000 Split, Croatia; helena456eric@gmail.com; 3 Department of Medical Laboratory Diagnostics, University Hospital of Split, 21000 Split, Croatia; daniela.supedomic@gmail.com; 4 Department of Health Studies, University of Split, 21000 Split, Croatia; 5 Department of Restorative Dental Medicine and Endodontics, Study of Dental Medicine, School of Medicine, University of Split, 21000 Split, Croatia

**Keywords:** oral lichen planus, serum calprotectin, oral lichen planus disease activity scale, C-reactive protein, erythrocyte sedimentation rate, lactate dehydrogenase

## Abstract

*Background and Objectives*: Oral lichen planus (OLP) is a mucocutaneous autoimmune disease with a complex aetiology and an entirely unknown pathophysiological mechanism. Calprotectin (CP) is an inflammation-related protein and a potential biomarker of disease activity. This study aimed to examine the association between serum calprotectin (sCP), disease duration, and disease activity in patients with erosive (active) and non-erosive (inactive) OLP. *Materials and Methods*: This cross-sectional study included 50 participants: 30 patients with OLP (20 erosive, 10 non-erosive) and 20 healthy controls. Medical and dental histories, sociodemographic data, disease duration, and OLP activity assessed by the Oral Lichen Planus Disease Activity Scale (OLP-DAS) were recorded, along with inflammatory markers [C-reactive protein (CRP), erythrocyte sedimentation rate (ESR), lactate dehydrogenase (LDH), and sCP]. *Results*: Non-erosive OLP patients had a statistically significantly higher sCP concentration than erosive patients (2.32 μg/mL vs. 1.34 μg/mL, *p* = 0.018). The area under the curve (AUC) was 43.9% in OLP patients (*p* = 0.470). There were no statistically significant differences between OLP patients and controls for ESR (*p* = 0.878), CRP (*p* = 0.439), or LDH (*p* = 0.476). There was a weak negative correlation between sCP concentration and disease duration in erosive (r_1_ = −0.115, p_1_ = 0.629) and non-erosive OLP (r_2_ = −0.166, p_2_ = 0.647). There was a moderate, negative, statistically significant correlation between sCP concentration and OLP-DAS (r = −0.455, *p* = 0.012). *Conclusions*: The findings of this study indicate that OLP inflammation is primarily local and chronic, rather than systemic. Serum CP showed limited diagnostic and prognostic value.

## 1. Introduction

Oral lichen planus (OLP) is a chronic inflammatory mucocutaneous disease of unknown aetiology, primarily affecting the oral mucosa, and is widely regarded as an immune-mediated disorder characterised by T-cell-mediated responses. OLP may present clinically as an isolated condition, while in the cutaneous form, oral lesions occur in up to 53.6% of patients [1,2]. The disease is characterised by unpredictable periods of exacerbation and remission, most commonly affecting the buccal mucous membranes, tongue, and gingiva [3]. The prevalence in the general population is 1.01%, with the highest rate in Europe at 1.43% and the lowest in India at 0.49% [4,5]. Epidemiological data indicate a higher incidence in women, particularly between the fourth and fifth decades of life [5,6]. The cause of OLP is not yet fully defined, but it involves a combination of genetic, immunological, and environmental factors [7,8]. The most common genetic associations include the human leukocyte antigen (HLA) alleles DR1, DR2, DR3, DR4, DR6, DR7, DR9, DRB1, DQA1, and DQB [2]. Specific forms of OLP have also been associated with HLAs. HLA-A3 is more frequently observed in patients with the reticular form of OLP, while HLA-A9 and B8 are associated with the erosive form [9]. Damage to the oral mucosa occurs through two main mechanisms: antigen-specific and non-specific activation of mast cells and matrix metalloproteinases (MMPs) [10,11]. Exogenous triggers include stress, certain dental restorative materials, and infectious agents such as Epstein–Barr virus (EBV), Varicella zoster virus (VZV), human herpesvirus (HHV) types 6 and 7, human papillomavirus (HPV), and hepatitis C virus (HCV). It often occurs with systemic comorbidities such as liver dysfunction, dyslipidaemia, diabetes mellitus (DM), hypertension, hypothyroidism, and inflammatory bowel disease (IBD) [2].

OLP presents in six distinct subtypes: reticular, papular, plaque, erosive/ulcerative, atrophic, and bullous. Clinically, OLP is commonly divided into non-erosive and erosive forms, with the latter representing a more severe phenotype characterised by symptomatic lesions and a potentially stronger association with systemic inflammatory responses [12]. Reticular, papular, and plaque forms are considered non-erosive (inactive), whereas erosive/ulcerative, atrophic, and bullous forms are classified as erosive (active). The main feature is the bilateral appearance of lesions, and it is not uncommon for several different clinical forms to occur simultaneously in one patient [13]. The World Health Organization (WHO) established the first standardised clinical and histopathological diagnostic criteria for OLP in 1978. These criteria were revised by van der Meij and van der Waal in 2003 [14], who proposed modified diagnostic criteria to improve specificity and distinguish OLP from oral lichenoid reaction (OLR). Further refinement was introduced by the American Academy of Oral and Maxillofacial Pathology (AAOMP) in 2016, which emphasised strict clinicopathological correlation and the importance of excluding epithelial dysplasia in the diagnosis of OLP [15]. According to the modified WHO criteria proposed by van der Meij and van der Waal, histopathological diagnosis is based on the presence of a well-defined, dense, band-like lymphocytic infiltrate in the superficial lamina propria accompanied by basal cell liquefaction degeneration, while the absence of epithelial dysplasia is required for a definitive diagnosis. In cases where histopathological features are not fully diagnostic, the term “histopathologically compatible with OLP” is recommended [14]. Histopathological diagnosis provides final confirmation for all forms of OLP except the reticular form, where the bilateral, symmetrical presence of Wickham’s striae is sufficient for diagnosis [16]. OLP is classified as an oral potentially malignant disorder (OPMD). The average risk of malignant transformation in OLP is 1.43%, but for specific clinical forms, such as erosive and ulcerative, this risk ranges from 1.0% to 10.0% [4,17].

Several scoring systems have been developed to assess the disease activity of OLP. However, only a few have been validated to ensure reproducible and accurate assessment. The main challenge is the diversity of clinical manifestations and the presence of lesions in different locations, which makes comprehensive assessment of disease activity difficult [18]. The most commonly used systems are the Thongprasom scoring system, the Oral Disease Severity Score (ODSS), the Reticular-Erythema-Ulcerative (REU) scoring system, and, most recently, the Oral Lichen Planus Disease Activity Scale (OLP-DAS) [19]. The OLP-DAS was developed to provide a comprehensive system for assessing the activity of OLP lesions, based on an extensive literature review and addressing the limitations of existing OLP assessment systems. It is currently the most up-to-date scale for evaluating the activity of oral lesions in OLP and is the result of collaboration among oral medicine experts from several Thai universities [20]. The content validity of the final OLP-DAS was evaluated by eight oral medicine specialists using a four-point Likert scale to assess its relevance and comprehensiveness. The reliability of the OLP-DAS was assessed in two contexts: inter-rater reliability and intra-rater reliability. The validity of the OLP-DAS was examined through convergent validity, for which two hypotheses were formulated: positive or at least moderate correlations between the total OLP-DAS scores and the total scores of other OLP severity assessment systems [ODSS and OLP-Investigator Global Assessment (OLP-IGA)], and positive or at least moderate correlations between the weighted disease activity scores according to the OLP-Severity Index (OLP-SI) component of the OLP-DAS and the scores of the ODSS subscale (ODSS activity score) [20]. A study was conducted to demonstrate the reliability and validity of the OLP-DAS in voluntary participants with a confirmed diagnosis of OLP and clinically present lesions. The results supported the reliability and validity of the OLP-DAS for use in patients with OLP [20]. The OLP-DAS offers significant advantages over other OLP scoring systems by reducing clinician subjectivity and eliminating the need to assess varying degrees of “redness”, a common source of variability. The scale also simplifies the assessment of complex lesions with a straightforward weighted scoring method, avoiding complicated calculations.

Calprotectin (CP) (S100A8/A9) is a calcium-binding heterodimeric protein in the S100 family, primarily located intracellularly in the cytoplasm of neutrophils, monocytes, and macrophages. It can constitute a significant proportion of cytosolic proteins and is involved in regulating the cytoskeleton and inflammatory responses. During cell activation or damage, S100A8/A9 is released into the extracellular space, either actively or passively, where it acts as a damage-associated molecular pattern (DAMP) and alarmin, exhibiting pronounced proinflammatory and antimicrobial effects [21]. CP plays a key role in innate immunity and the recruitment of inflammatory cells, serving as an important marker of inflammation. It is produced by activated immune cells, such as neutrophils and monocytes, as well as by endothelial cells. Its levels rise rapidly in the presence of microorganisms, including bacteria, due to its direct bactericidal properties mediated by the chelation of magnesium and zinc ions [22]. CP concentration is measured in serum (sCP) and stool (fCP) samples. Reference values for healthy individuals are typically below 1 μg/mL for sCP and below 50 μg/g for fCP. In clinical practice, sCP shows greater specificity in diagnosing autoimmune diseases, while fCP is notable for its high sensitivity and specificity in distinguishing active forms of IBD from irritable bowel syndrome (IBS) [22]. Recent studies indicate that sCP correlates more closely with inflammatory disease activity than traditional biomarkers such as C-reactive protein (CRP) or erythrocyte sedimentation rate (ESR) [23]. Given the key role of CP in the pathophysiology of acute and chronic inflammation, sCP levels are a promising biomarker for assessing autoimmune disease activity [23].

The main objective of this study was to compare sCP concentrations between patients with erosive (active) and non-erosive (inactive) forms of OLP. The specific objectives were to assess the sociodemographic, clinical, and laboratory characteristics of all subjects, determine disease duration in patients with OLP, and evaluate disease activity using the OLP-DAS. The hypothesis was that patients with erosive (active) OLP have higher sCP concentrations than those with non-erosive (inactive) disease, and that sCP concentrations are associated with disease duration and disease activity.

## 2. Materials and Methods

### 2.1. Study Design, Subjects, Inclusion and Exclusion Criteria

This cross-sectional study included 50 subjects from the Department of Dental Medicine, University Hospital of Split, Split, Croatia, divided into two groups: 1. Patients with a clinically and histopathologically confirmed diagnosis of OLP according to the modified WHO criteria proposed by van der Meij and van der Waal (2003) (N = 30) [14]; 2. Healthy control subjects (N = 20). The OLP group comprised 20 patients with the erosive (active) form and 10 with the non-erosive (inactive) form. OLP patients were classified as erosive (active) or non-erosive (inactive) based on clinical presentation. According to commonly accepted clinical classification, reticular, papular and plaque forms are considered non-erosive (inactive), whereas erosive/ulcerative, atrophic and bullous forms are classified as erosive (active). The erosive (active) forms are typically symptomatic (pain, burning), while the non-erosive (inactive) forms are usually asymptomatic [2,24]. There is no comprehensive diagnostic tool to distinguish between the different clinical forms of OLP [14]. Dividing OLP patients into erosive (active) and non-erosive (inactive) forms is essential for understanding their biological characteristics [25]. In this study, the prevalence of these two forms is unequal, which is consistent with the clinical presentation of the disease, as the erosive form is less common [1].

Control subjects were recruited from the same clinical setting at the Department of Dental Medicine, University Hospital of Split, Split, Croatia. They were selected by simple random sampling. All eligible healthy individuals were assigned a numerical code, and a random number generator was used to select participants to minimise selection bias. There were no statistically significant differences in age or sex distribution between the groups.

The inclusion criteria for OLP patients were:

1. Clinically and histopathologically confirmed diagnosis of OLP according to the modified WHO criteria proposed by van der Meij and van der Waal (2003) [14];
2. Age ≥ 18 years.

The exclusion criteria for OLP patients were:

1. Presence of systemic diseases or conditions (DM, cardiovascular diseases, renal dysfunction, liver diseases, autoimmune diseases, cancers);
2. Use of medications such as corticosteroids, immunosuppressants, non-steroidal anti-inflammatory drugs (NSAIDs), biologics, disease-modifying antirheumatic drugs (DMARDs), antibiotics, granulocyte colony-stimulating factor (G-CSF), or lithium;
3. Harmful habits, including smoking or chewing betel nut or tobacco;
4. Presence of cutaneous lichen planus (LP).

The exclusion criteria for control subjects were:

1. Oral mucosal diseases or systemic diseases;
2. Smoking in any form;
3. Age < 18 years.

### 2.2. Ethical Approval and Study Protocol

This study was conducted at the Department of Dental Medicine, University Hospital of Split, Split, Croatia, from April to September 2025. The study received approval from the Ethics Committee of the University Hospital of Split, Split, Croatia (Class: 520-03/25-01/59, Reg. No.: 2181-147/01-06/LJ.Z.-25-02) on 20 February 2025. All subjects participated voluntarily and their data were pseudonymised. They received detailed information about the protocol and purpose of the study, both in writing and orally, before signing the informed consent form.

A medical and dental history was obtained from all subjects, and sociodemographic data (age, gender), disease duration (months), disease activity in OLP patients (using OLP-DAS), and values of standard inflammatory parameters [CRP, ESR, lactate dehydrogenase (LDH), sCP] were recorded. The same oral medicine specialist (A.G.), with more than five years of specialist experience, conducted a clinical oral examination of all subjects.

### 2.3. Serum CP Sampling

Serum CP concentrations were determined from blood serum obtained by centrifuging venous blood. Samples were collected at the Department of Dental Medicine, University Hospital of Split, Split, Croatia and then analysed at the Department of Medical Laboratory Diagnostics, University Hospital of Split, Split, Croatia.

Quantitative determination of sCP concentrations was performed using B-KSCAL-R1 and B-KSCAL-R2 reagents (Bühlmann Laboratories, Schönenbuch, Switzerland) with a particle-enhanced turbidimetric immunoassay (PETIA) on the Abbott Alinity biochemical analyser (Abbott Laboratories, Wiesbaden, Germany). Samples were measured without further dilution. They were incubated with reaction buffer and mixed with polystyrene nanoparticles coated with antibodies specific to CP. The turbidity of the sample, resulting from agglutination of the immunoparticles and CP, was monitored spectrophotometrically. The measuring range of the assay was 0.3–15 µg/mL (extended range up to 225 µg/mL). The turbidity, measured as light absorbance, is directly proportional to the sCP concentration. The detected absorbance enabled quantification of CP concentration by interpolation on the established calibration curve. Results were automatically calculated on the biochemical analyser and presented in µg/mL.

### 2.4. Instruments

#### OLP-DAS

The OLP-DAS was used to assess lesion activity. The OLP-DAS score is the sum of the total OLP-Sign Score (OLP-SS; 0–65), the OLP-SI (0–20), and the numerical rating scale (NRS; 0–10) for the worst pain experienced in the past week. The total range of the OLP-DAS score is 0–95 [20]. The OLP-SS, a modification of Thongprasom’s criteria, considers the clinical appearance of the lesion and the affected area in each oral region [20]. The addition of the OLP-SI component provides weighted scores for more oral sites with erythema (OLP-SS = 2–3) and ulceration (OLP-SS = 4–5), allowing a more accurate assessment of disease activity [20]. Pain intensity is assessed using the NRS, an 11-point scale ranging from 0 (no pain) to 10 (worst pain imaginable), based on pain experienced in the past week [20]. The primary purpose of the OLP-DAS scale is to monitor disease dynamics, not to classify the disease. The initial score serves as a reference point. A decrease in the score indicates therapeutic success and improvement in the patient’s condition, while an increase suggests disease progression or an inadequate response to treatment [20]. The OLP-DAS provides a comprehensive overview of the patient’s condition by covering three key areas: lesion appearance, severity, and patient-reported symptoms [20].

### 2.5. Sample Size

An a priori power analysis was conducted using the G*Power programme (version 3.1.9.7; F-test, Analysis of Variance (ANOVA): fixed effects, omnibus, one-way) to determine the required sample size [26]. The analysis assumed an effect size of f = 0.52, a significance level of α = 0.05, statistical power (1 − β) = 0.95, and three groups. The power analysis indicated that a total sample size of N = 53 participants was required, corresponding to approximately 17–18 participants per group, assuming equal group sizes.

The final sample comprised 50 participants: 30 patients with OLP (20 with erosive and 10 with non-erosive forms) and 20 control subjects, based on the availability of eligible participants during the study period. The unequal group sizes reflect the natural distribution and recruitment feasibility of specific clinical subtypes, particularly the lower prevalence of the non-erosive form. Nevertheless, one-way ANOVA is considered robust to moderate deviations from equal group sizes, especially when group variances are comparable. A post hoc power analysis indicated that the achieved statistical power for the final sample size of 50 participants was approximately 0.89.

### 2.6. Statistical Analysis

Statistical analysis was performed using IBM SPSS Statistics version 23.0 (IBM Corp., Armonk, NY, USA). The normality of data distribution was assessed with the Shapiro–Wilk test, and homogeneity of variances with Levene’s test. Categorical variables are presented as frequencies and percentages. Continuous variables are presented according to their distribution: mean ± standard deviation (SD) for normally distributed data, or median with interquartile range (IQR) for non-normally distributed data.

For group comparisons, one-way ANOVA was used for normally distributed variables, with the Scheffé post hoc test applied when appropriate to adjust for multiple comparisons and control the type I error rate. For non-normally distributed variables, the Kruskal–Wallis test was used. Correlations between continuous variables were assessed using Pearson’s correlation coefficient for normally distributed data and Spearman’s rank correlation coefficient for non-normally distributed data. A *p*-value < 0.05 was considered statistically significant.

## 3. Results

### 3.1. Clinical and Laboratory Parameters

A total of 50 subjects participated in the cross-sectional study, divided into two groups: OLP patients and controls. The clinical and demographic characteristics of OLP patients are summarised in Table 1.

OLP patients were divided into two subgroups based on clinical form: erosive (active) (N = 20) and non-erosive (inactive) (N = 10). The control group comprised 20 healthy subjects. Of all participants, 80% (N = 40) were women. There was no statistically significant difference between the groups in gender (*p* = 0.596) or age (*p* = 0.250). Among the laboratory inflammatory parameters, there was no statistically significant difference between the OLP and control groups for ESR (*p* = 0.878), CRP (*p* = 0.439), or LDH (*p* = 0.476). However, there was a statistically significant difference in sCP concentration between the three groups (*p* = 0.017) (Table 2).

**Figure 1:**
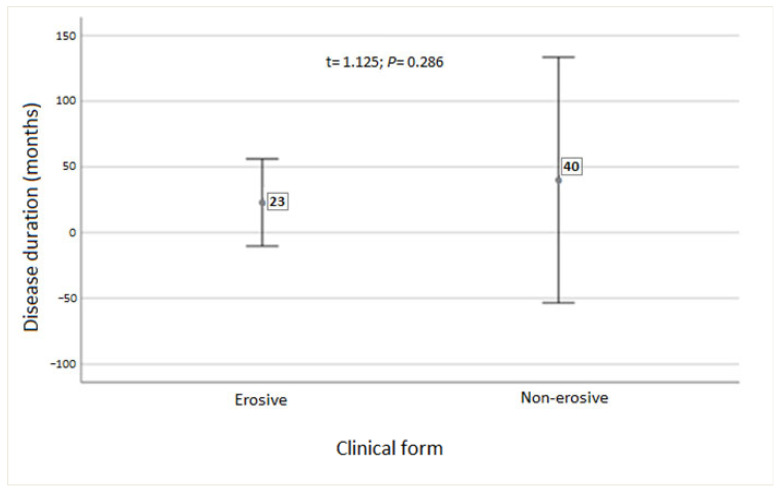
Mean disease duration in erosive (active) and non-erosive (inactive) forms of OLP. Data are presented as arithmetic mean and confidence interval. *t*-test for independent samples.

The mean disease duration was 23 months for erosive (active) OLP and 40 months for non-erosive (inactive) OLP. There was no statistically significant difference in disease duration between the OLP groups (*p* = 0.286; see Figure 1).

### 3.2. Receiver Operating Characteristic (ROC) Curve for Serum CP

**Figure 2:**
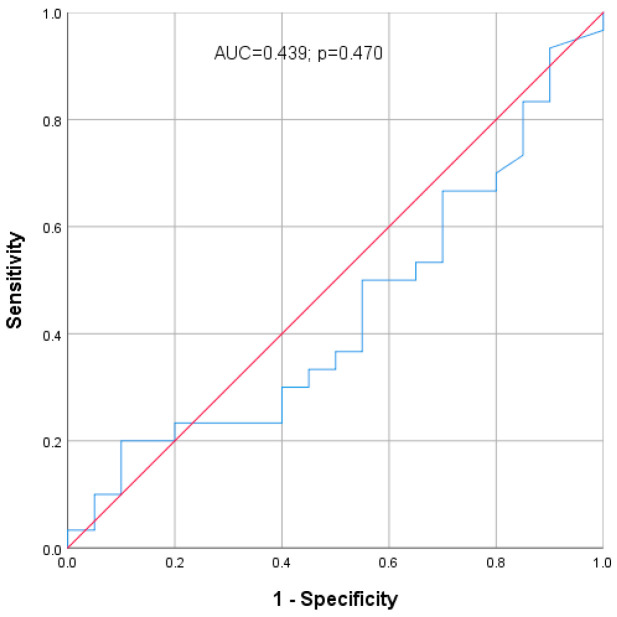
ROC curve for sCP concentration in patients with OLP. The blue line represents the ROC curve, while the red diagonal line indicates the reference line of no discrimination. Abbreviations: AUC, area under the ROC curve; ROC, receiver operating characteristic curve; sCP, serum calprotectin; OLP, oral lichen planus.

The ROC analysis of sCP concentration is shown in Figure 2. The area under the ROC curve (AUC) was 0.439 (43.9%), indicating insufficient discriminatory ability; the AUC was not statistically significant (*p* = 0.470). Therefore, sCP concentration did not demonstrate a useful ability to discriminate between the groups.

### 3.3. Serum CP Concentration

**Figure 3:**
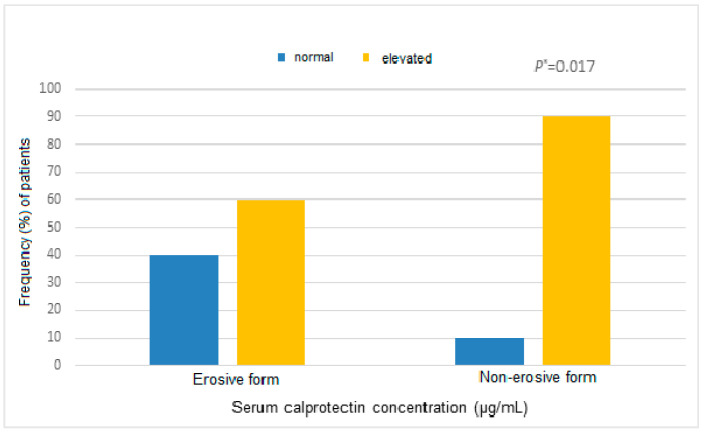
Distribution of sCP levels in patients with erosive (active) and non-erosive (inactive) forms of OLP. “Normal” indicates concentrations below the cut-off value, while “elevated” indicates concentrations above the cut-off value. Data are presented as frequency (%). Statistical significance was assessed using the chi-square test or Fisher’s exact test, as appropriate. * *p* value indicates statistical significance. Abbreviations: sCP, serum calprotectin; OLP, oral lichen planus;.

Patients with the non-erosive (inactive) form of OLP had a statistically significantly higher sCP concentration than those with the erosive (active) form (2.32 vs. 1.34, *p* = 0.018) (Figure 3).

There was a weak, negative, statistically significant correlation between sCP concentration and LDH (see Table 3).

**Figure 4:**
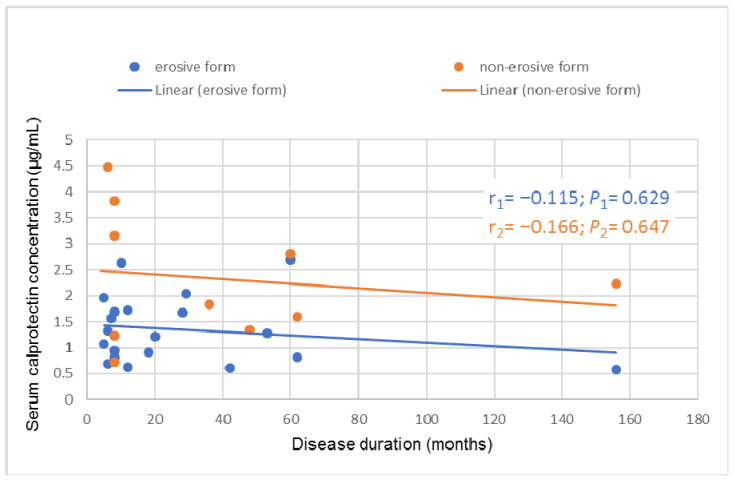
Correlation between disease duration and sCP concentration in patients with erosive (active) and non-erosive (inactive) forms of OLP. Pearson correlation coefficient (r). Abbreviations: sCP, serum calprotectin; OLP, oral lichen planus.

There was a weak negative correlation between sCP concentration and disease duration in patients with erosive (active) (r_1_ = −0.115, p_1_ = 0.629) and non-erosive (inactive) (r_2_ = −0.166, p_2_ = 0.647) forms of OLP (Figure 4).

**Figure 5:**
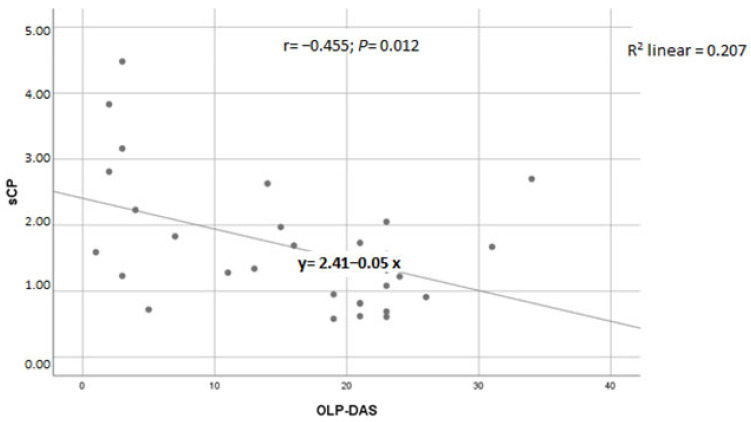
Correlation between sCP concentration and OLP-DAS score in patients with OLP. Pearson’s correlation coefficient (r). Simple linear regression analysis showed R^2^ = 0.207 (y = 2.41 − 0.05 x). Abbreviations: sCP, serum calprotectin; OLP-DAS, oral lichen planus disease activity scale; OLP, oral lichen planus.

There was a moderate, negative, statistically significant correlation between sCP concentration and OLP-DAS (r = −0.455, *p* = 0.012; Figure 5).

## 4. Discussion

The aim of this cross-sectional study was to determine and compare sCP concentrations in patients with OLP and healthy controls. The specific aims were to assess whether sCP concentrations differ according to the clinical form of the disease (erosive or non-erosive) and to evaluate the potential role of sCP in assessing OLP disease activity. The results did not support the hypothesis. Serum CP concentrations did not correlate with disease duration or OLP disease activity as assessed by the OLP-DAS.

Analysis of sCP concentrations showed a statistically significant difference between erosive (active) and non-erosive (inactive) forms of OLP, with higher concentrations observed in the non-erosive (inactive) group. However, further analysis indicated that this difference was insufficient to support the use of sCP for monitoring disease activity in OLP. There was considerable overlap in sCP concentrations between groups, suggesting limited discriminatory capacity. The unexpected finding of higher sCP concentrations in patients with non-erosive OLP compared to those with erosive OLP may be explained by disease heterogeneity, as clinical classification does not always reflect underlying inflammatory activity. Subclinical inflammation may persist in clinically inactive lesions. Furthermore, the cross-sectional design may not capture temporal variations in inflammatory markers. Methodological factors, including sample size and inter-individual variability, may also have influenced the results. A literature search found no directly comparable studies investigating sCP concentrations in OLP making direct comparison with previous findings impossible. The study by Saviano A et al. (covering studies from 2014 to 2024), although focused on gastrointestinal (GI) diseases, reported that sCP concentration may reflect local inflammatory activity and correlate with clinical indices and endoscopic findings, suggesting a potential role as a systemic marker of local inflammation [22].

As previously stated, our study does not provide sufficient evidence to support the use of sCP as a biomarker for assessing disease activity in OLP. One possible explanation is the predominantly local and chronic nature of inflammation in OLP. This interpretation is consistent with the observation that other systemic inflammatory parameters (CRP, ESR, LDH) were within reference values in most patients. The absence of a statistically significant correlation between total sCP concentration and disease duration may also be related to the localised and fluctuating nature of OLP, characterised by periods of remission. Our findings are consistent with the study by Družijanić A et al., which included 63 OLP patients and 63 control subjects without pathological changes in the oral mucosa. In all subjects, three systemic inflammatory parameters were determined: ESR, CRP, and leukocyte count (Lkc) from a blood sample. No statistically significant difference was found between OLP patients and the control group for any of the observed systemic inflammatory parameters (*p* = 0.364 for ESR; *p* = 1.000 for CRP; *p* = 0.219 for Lkc) [27]. In contrast, studies examining systemic non-specific inflammatory parameters such as interleukin 6 (IL-6) have found increased levels in OLP patients. This is supported by a meta-analysis by Mozaffari HR et al., which included 11 studies (529 OLP patients and 333 healthy controls) investigating serum and salivary IL-6 concentrations in OLP patients. A pooled analysis of seven studies showed a statistically significantly higher serum IL-6 concentration compared to healthy controls (*p* = 0.002), while salivary IL-6 concentrations were even higher (*p* = 0.001) [28]. Anwar R et al. conducted a review up to 2022 on the role of salivary CP in disease activity in OLP. Their results showed statistically significantly higher salivary CP concentrations in OLP patients, suggesting its role as a local biomarker of inflammation and as a potential diagnostic and prognostic tool [29]. Although the role of sCP in reflecting local inflammation in OLP remains unclear, the findings of this study suggest it has limited utility. Numerous studies emphasise its key role in systemic inflammatory processes, while in the context of local oral inflammation, salivary CP may be more appropriate.

OLP lesion activity was assessed using the OLP-DAS tool. In this study, OLP-DAS scores served as a clinical measure of disease activity. A moderate, statistically significant negative correlation was found between OLP-DAS scores and sCP concentration. However, this association was not strong enough to support sCP as an independent inflammatory biomarker for assessing OLP activity, suggesting that its concentrations may be influenced by additional, as yet unidentified, factors. Further longitudinal studies are needed to clarify the relationship between OLP-DAS and inflammatory biomarkers in OLP.

Our study contributes to understanding the potential role of sCP as a biomarker in OLP. The findings do not provide sufficient evidence to support its use as a marker of local inflammatory activity in OLP and highlight the need for further research. Previous studies have demonstrated the relevance of sCP in the diagnosis and monitoring of systemic inflammatory processes. Therefore, it is important to conduct additional targeted research to determine whether this biomarker can play a more significant role in specific conditions of OLP. These findings underscore the need for further investigation into the relationship between local oral inflammation and the systemic inflammatory response, which may lead to a better understanding of disease mechanisms and the identification of potential biomarkers in OLP.

The strengths of our study include the use of sCP concentration as a potential biomarker of inflammation and, to our knowledge, one of the first applications of this approach in patients with OLP. The OLP-DAS was also used as a clinical measure of disease activity. This study has several limitations that should be considered in future research. The sample size was relatively small and uneven across groups, and data were collected at a single centre. Therefore, multicentre studies with larger sample sizes are needed to improve generalisability. As this was a cross-sectional study, longitudinal research is required to better understand disease dynamics and treatment response.

## 5. Conclusions

Although patients with non-erosive (inactive) OLP had statistically significantly higher sCP concentrations than those with the erosive (active) form, sCP did not provide sufficient evidence to serve as a biomarker for monitoring disease activity in OLP. This may reflect the predominantly local and chronic nature of inflammatory processes in OLP. Therefore, sCP alone does not appear suitable as a diagnostic or prognostic biomarker. Other systemic inflammatory parameters (CRP, ESR, LDH) did not differ statistically significantly between the three groups, further supporting the local nature of OLP inflammation. No correlation was found between sCP concentration and disease duration or activity. In contrast, OLP-DAS, used as a clinical measure of disease activity, showed an association with clinical disease status.

## Figures and Tables

**Table 1:** Clinical and demographic characteristics of OLP patients.

Variable	OLP Patients (N = 30)
Age (years)	59.20 *±* 14.42
Sex (M:F)	6 (20.0%)/24 (80.0%)
Disease duration (months)	20.5 (8.0–36.5)
OLP-DAS	18.0 (6.0–23.0)

Data are presented as mean ± standard deviation or median (interquartile range), as appropriate. Abbreviations: OLP, oral lichen planus; M, male; F, female; OLP-DAS, oral lichen planus disease activity scale.

**Table 2:** Comparison of sociodemographic characteristics and laboratory parameters between the groups.

Parameter	Erosive OLP Group (N = 20)	Non-Erosive OLP Group (N = 10)	Control Group (N = 20)	*p*
Age (years)	60.3 ± 12.29	52.1 ± 18.01	58.9 ± 10.1	0.250 *
Sex ratio (M:F)	1:5.67	1:2.33	1:4	0.596 *
sCP (μg/mL)	1.34 ± 0.64	2.32 ± 1.22	1.75 ± 0.82	0.017 ^†^
ESR (mm/h)	9.0 (11)	10.5 (12)	12.5 (11)	0.878 ^‡^
CRP (mg/L)	1.15 (1.35)	1.95 (1.7)	1.35 (1.48)	0.439 ^‡^
LDH (U/L)	181.6 ± 27.93	169.3 ± 24.31	175.15 ± 26.47	0.476 *

Data are presented as numbers (percentages), arithmetic mean ± standard deviation, or median (interquartile range). * One-way analysis of variance (ANOVA). † ANOVA with post hoc Scheffé test. ‡ Kruskal–Wallis test. Post hoc Scheffé test—sCP: Erosive vs. Non-erosive: *p* = 0.018; Erosive vs. Control: *p* = 0.327; Non-erosive vs. Control: *p* = 0.233. Abbreviations: M, male; F, female; sCP, serum calprotectin; ESR, erythrocyte sedimentation rate; CRP, C-reactive protein; LDH, lactate dehydrogenase; OLP, oral lichen planus; N, number.

**Table 3:** Correlation between sCP and laboratory inflammatory parameters.

Parameter	sCP	
	ρ	*p*
ESR	−0.099	0.493
CRP	0.107	0.461
LDH	−0.315	0.026

Spearman’s rank correlation coefficient (ρ). Correlation is significant at *p* < 0.05. Abbreviations: sCP, serum calprotectin; ESR, erythrocyte sedimentation rate; CRP, C-reactive protein; LDH, lactate dehydrogenase.

## Data Availability

The data analysed in this study are available upon reasonable request by email to the corresponding author.

## Abbreviations

The following abbreviations are used in this manuscript:

OLP	Oral lichen planus
CP	Calprotectin
sCP	Serum calprotectin
OLP-DAS	Oral Lichen Planus Disease Activity Scale
CRP	C-reactive protein
ESR	Erythrocyte sedimentation rate
LDH	Lactate dehydrogenase
AUC	Area under the curve
HLA	Human leukocyte antigen
MMP	Matrix metalloproteinase
EBV	Epstein–Barr virus
VZV	Varicella zoster virus
HHV	Human herpesvirus
HPV	Human papillomavirus
HCV	Hepatitis C virus
DM	Diabetes mellitus
IBD	Inflammatory bowel disease
WHO	World Health Organization
AAOMP	American Academy of Oral and Maxillofacial Pathology
OLR	Oral lichenoid reaction
OPMD	Oral potentially malignant disorder
ODSS	Oral Disease Severity Score
REU	Reticular-Erythema-Ulcerative
DAMP	Damage-associated molecular pattern
fCP	Faecal calprotectin
IBS	Irritable bowel syndrome
NSAID	Non-steroidal anti-inflammatory drug
DMARD	Disease-modifying antirheumatic drug
G-CSF	Granulocyte colony-stimulating factor
LP	Lichen planus
PETIA	Particle-enhanced turbidimetric immunoassay
OLP-IGA	Oral Lichen Planus-Investigator Global Assessment
OLP-SI	Oral Lichen Planus-Severity Index
OLP-SS	Oral Lichen Planus-Sign Score
NRS	Numerical rating scale
ANOVA	Analysis of variance
SD	Standard deviation
IQR	Interquartile range
N	Number
M	Male
F	Female
ROC	Receiver Operating Characteristic curve
GI	Gastrointestinal
Lkc	Leukocyte
IL-6	Interleukin 6
